# The impact of physical exercise on mobile phone addiction among college students: a study based on Chinese universities

**DOI:** 10.3389/fpsyg.2025.1524520

**Published:** 2025-04-14

**Authors:** Bo Shi, Di Wang, Mengfan Liu

**Affiliations:** ^1^School of Physical Education, Northeast Normal University, Changchun, China; ^2^School of Psychology, Northeast Normal University, Changchun, China

**Keywords:** physical exercise, mobile phone addiction, college students, questionnaire survey, empirical analysis

## Abstract

To explore the impact of physical exercise on mobile phone addiction (MPA) among college students and the underlying psychological mechanisms, a questionnaire survey method is utilized to conduct empirical analysis on students from multiple colleges in China. This study uses Physical Activity Rating Scale-3, Mobile Phone Addiction Tendencies Scale (MPATS), Self-Acceptance Questionnaire, and Chinese Perceived Stress Scale to measure psychological variables such as physical activity level, degree of MPA, and self-acceptance and perceived stress among college students. The study reveals the mechanism of physical exercise in reducing MPA. The research results demonstrate a significant negative correlation between MPA and physical exercise. Exercise frequency has the most remarkable influence on withdrawal symptoms and salience behaviors, with correlation coefficients of −0.35 and −0.30, respectively. These findings show that regular engagement in physical activity can substantially mitigate the dependency of college students on mobile phones. Exercise intensity and exercise duration also have a negative effect on MPA, and the correlation coefficient of exercise intensity on withdrawal symptoms is −0.32. Furthermore, self-acceptance as a moderating variable plays an important protective role within the nexus between physical exercise and MPA. Students with higher self-acceptance levels show significant remission in both withdrawal symptoms (*β* = −0.30) and mood changes (*β* = −0.28), with model interpretability increasing from 0.34 to 0.43. On the contrary, perceived stress, as a risk-regulating variable, is positively correlated with MPA, and the impact of perceived stress on withdrawal symptoms is 0.35. Additionally, under conditions of elevated perceived stress, the physical exercise’s mitigating effect on mobile phone dependence is attenuated. Therefore, self-acceptance can enhance the individual’s self-identity, weaken the negative emotional reaction brought by MPA, and help to improve the intervention effect of physical exercise. On the contrary, perceived stress weakens the relieving effect of physical exercise on mobile phone dependence, and individuals with high-stress levels are more likely to maintain MPA behavior. This study makes a valuable contribution to the literature on the interplay.

## 1 Introduction

With the swift advancement of mobile Internet technology, smartphones have emerged as an essential component of the daily lives of modern college students. Through mobile phones, college students not only access a large amount of information and engage in social interaction but also improve their learning efficiency through various applications (Guo et al., 2022). However, the pervasive adoption of smartphones has introduced adverse effects, with the issue of Mobile Phone Addiction (MPA) escalating notably among college students, becoming one of the critical factors affecting their learning, mental health (MH), and social abilities (Mei et al., 2023). As per the 48th Statistical Report on Internet Development in China, published by the China Internet Network Information Centre, by the conclusion of 2021, the populace of Internet users in China had surged to 1.032 billion. Within this demographic, 1.029 billion were mobile Internet users, constituting an overwhelming 99.7% of the entire user base. As the main mobile Internet users, college students’ dependence on mobile phones has aroused widespread concern (Xiao et al., 2022; Liu et al., 2022). Excessive use of mobile phones can lead to distraction and decreased academic performance. It can even trigger MH problems such as depression and anxiety. Therefore, effectively reducing MPA has emerged as a significant concern within the realms of college education and society. Concurrently, physical exercise has garnered escalating interest as a potent means to bolster college students’ physical and MH (Lin et al., 2022). Numerous studies have shown that physical activities enhance physical fitness and remarkably improve MH, reduce stress, and alleviate anxiety and depression. The World Health Organization has consistently underscored the salutary effects of physical exercise in preventing and treating MH issues. However, research pertaining to the influence of physical exercise on MPA behavior among college students is currently scarce, particularly within the context of Chinese universities, where such inquiries remain relatively uncharted.

In this context, this study explores the physical exercise’s influence on MPA among college students, particularly analyzing the regulatory role of physical exercise in mitigating mobile phone dependence behavior. By combining psychological variables such as mobile phone dependence, self-acceptance, physical activity level, and perceived stress, this study delves into the regulatory mechanism of physical exercise on college students’ MH, to provide a reference for health education in universities.

## 2 Literature review

To comprehensively explore the physical exercise’s impact on college students’ MPA, this study divides the literature review into three dimensions, encompassing the interplay between MPA and MH, and the influence of physical exercise on MPA behavior and MH.

In terms of the influence of physical exercise on MH, Liu and Shi (2023) studied the physical exercise’s effect on college students’ MH. They used the Generalized Anxiety Disorder (GAD-7) and the Physical Activity Rating Scale (PARS)-3 to study the correlation between the frequency and intensity of physical exercise and anxiety. The findings found that regular moderate-intensity physical exercise could significantly reduce anxiety in college students, and improve their overall MH. They believed that physical exercise could improve the psychological state by stimulating the body’s positive emotions and reducing negative emotions. It was suggested that college students should maintain regular physical exercise to relieve learning pressure and emotional distress (Liu and Shi, 2023). Lu et al. (2022) deeply examined the mechanism of emotional regulation through physical exercise. In the experiment, 100 college students were selected as samples and divided into control and experimental groups. The experimental group received a 12-week aerobic exercise intervention. The results showed that students who participated in physical exercise exhibited prominent improvements in emotional regulation, stress coping, and psychological resilience. Furthermore, they pointed out that physical exercise not only alleviated individuals’ anxiety and depression but also enhanced their emotional control ability, thereby improving psychological resilience (Lu et al., 2022). Zhang D. et al. (2022) investigated the differential effects of various physical activities on MH. They compared the different effects of aerobic exercise (such as running and swimming) and anaerobic exercise (such as weightlifting and strength training) on emotions and cognitive function. They discovered that aerobic exercise had a more significant effect on improving emotions and reducing anxiety and depression, while anaerobic exercise improved individuals’ self-efficacy and cognitive performance more. Their research further revealed the multidimensional impacts of physical exercise on MH, emphasizing that diverse types of exercise had targeted effects on various psychological problems (Zhang D. et al., 2022). Existing studies generally show that physical exercise, especially regular and moderate intensity exercise, plays a significant role in relieving anxiety, and improving emotional regulation ability and overall MH of college students. At the same time, different types of physical activity have differentiated positive effects on psychological state, reflecting the multi-dimensional value of physical exercise in psychological intervention.

Zhang et al. (2023) studied the longitudinal relationship between MPA and sleep disorders (SD) in Chinese college students. They used the Smartphone Addiction Scale - Short Version (SAS-SV) and the Pittsburgh Sleep Quality Index (PSQI) to collect data at intervals of 1 year. The results showed that there was a two-way relationship between MPA and SD, but this effect disappeared in people who exercised more than 1 h a day. This not only means that MPA can lead to psychological problems and SD, but also suggests that exercise may be an extremely effective intervention (Zhang et al., 2023). Achangwa et al. (2022) studied the impact of MPA on the academic performance of college students. They found through a questionnaire survey and academic performance analysis of 500 college students that MPA not only affected students’ classroom attention and learning efficiency but also had a negative correlation with academic performance. Research showed that students with severe MPA often had difficulty concentrating on completing learning tasks and were more prone to avoid real-life problems. They called on schools to take measures to reduce the negative impact of mobile phones on students’ learning (Achangwa et al., 2022). It can be found that previous studies have shown that MPA of college students not only has a two-way correlation with SD but also has a significant negative impact on academic performance. However, regular physical exercise can effectively alleviate this negative effect, suggesting that exercise intervention has a vital value in improving the psychological and learning problems caused by MPA in college students.

Considering the impact of physical exercise on MPA behavior, Du and Zhang (2022) revised PARS-3 in their study and explored the effects of physical activity on psychological stress and addictive behavior among college students. Their research found that students with higher physical activity levels exhibited stronger adaptability in coping with academic and social pressures. Concurrently, their Mobile Phone Addiction Tendency (MPAT) was remarkably lower than that of students who did not participate in physical activities. The research results revealed that physical exercise could help improve students’ MH status and effectively reduce their dependence on mobile phones (Du and Zhang, 2022). Zhou and Wang (2022) used the antisaccade task to test the effects of aerobic exercise on inhibitory control of college students with MPA. In the experiment, participants were required to perform 15 min of aerobic exercise, and antisaccade tasks were used to check inhibition control before and after exercise. The results showed that participants “inhibitory control ability was significantly stronger after exercise, so they believed that aerobic exercise could indeed improve MPA (Zhou and Wang, 2022). Yang et al. (2021) studied the physical exercise type’s regulating effect on college students” MPA. They used the mobile phone addiction tendency scale (MPATS) and the Physical Activity Rating Scale (PARS-3) to study the correlation between the type, frequency and intensity of physical exercise and MPA. The findings found that physical exercise was an imperative protective factor to decrease MPA, from low exercise to medium exercise, MPA gradually decreased. More importantly, the type of exercise can play a regulatory role. Specifically, the more aerobic exercise college students have, the lower their MPA is (Yang et al., 2021). Thus, it is generally believed that physical exercise plays a positive role in reducing college students’ MPA. This helps to relieve psychological pressure and improve inhibitory control ability. Meanwhile, the frequency, intensity, and type of exercise (especially aerobic exercise) play a critical protective role in regulating MPA behavior.

It can be found that many existing studies mainly focus on specific regions or schools, and the representation of samples is relatively weak, which makes it difficult to fully reflect the current situation of MPA among college students nationwide. For example, some studies only select students from one university or a certain region as samples, and the results are not universally applicable, which poses a challenge for promoting to Chinese college students. Moreover, most studies primarily employ cross-sectional questionnaire surveys and correlation analysis. Although it can reveal the relationship between physical exercise and MPA, few studies use experimental design or longitudinal tracking methods to verify the causal relationship. For instance, although studies have exhibited a certain negative correlation between physical exercise and MPA, whether physical exercise can effectively reduce MPA behavior has not been validated through rigorous experimental design. The research object of this paper covers many universities, has a relatively wide geographical distribution, and the sample representation is stronger. This design facilitates drawing more generalizable conclusions and improving the external validity of the findings.

## 3 Research design

### 3.1 Questionnaire design

To comprehensively investigate the current situation and interplay between physical exercise and MPA of college students, this study designs a multidimensional questionnaire. It aims to cover multiple important variables such as basic information, physical activity, MPA behavior, self-acceptance level, and perceived stress of the participants (Abbasi et al., 2021; Niu, 2023). The questionnaire design strictly follows scientific principles, refers to mature scales in relevant fields, and makes appropriate adjustments based on actual situations to guarantee the data validity and reliability.

The questionnaire is divided into five parts, and the design of the basic information part is majorly used to collect the personal basic information of the participants, helping researchers to statistically describe and classify the sample. This section includes information such as gender, grade level, and family location. Gender is employed to analyze the impact of gender differences on physical exercise and MPA (Haripriya et al., 2022; Numanoğlu-Akbaş et al., 2020; Jin et al., 2024). Grades are utilized to explore the differences in mobile phone usage habits and exercise frequency among students at diverse learning stages through the division of different grades (Wan and Ren, 2023). The family location is applied to analyze the impact of urban–rural differences on the MPA and physical activity habits of college students. This information provides pivotal background variable support for subsequent data analysis, which can help researchers consider the influence of factors such as gender, grade, and region in their analysis. The other four parts are the Chinese Perceived Stress Scale (CPSS), Self-Acceptance Questionnaire (SAQ), PARS-3, and MPATS. There were 54 questions in the questionnaire. These scales are mature questionnaire tools widely used domestically and internationally, with good theoretical basis and application experience. Therefore, the questionnaire design in this study is based on a combination of mature questionnaires, and its reliability and validity have been verified in a large number of studies. Moreover, this study does not repeat the reliability and validity analysis.

### 3.2 Sample selection and data collection

#### 3.2.1 Sample selection

The target group of this study is Chinese college students. To ensure the research results’ wide applicability and external validity, this study adopts a stratified random sampling method, ensuring that the sample has high representativeness in terms of gender, grade, region, etc. (Zhang A. et al., 2022).

1. Gender distribution: To avoid gender bias, the sample selection process strictly ensures a balanced gender ratio. According to statistical data, the gender ratio in Chinese universities is generally close to equilibrium. Therefore, this study plans to select about 50% males and 50% females as participants. This ensures that the differences between men and women in physical exercise, MPA, and other aspects can be fairly reflected in subsequent data analysis (Suman and Devasirvadam, 2022).
2. Grade distribution: this study covers students from freshman to senior year, aiming to analyze the changes in physical exercise habits and mobile phone usage behavior among students at different learning stages. Due to significant differences in academic pressure and pace of life among students of diverse grades, it is necessary to ensure a balanced distribution of grades in the sample. In the planned sample, freshmen to seniors account for about 25% of the total sample respectively, to guarantee that the physical exercise behavior and MPA of various grade groups can be comprehensively reflected (Huang et al., 2022).
3. Regional distribution: the source of students in Chinese universities has strong regional diversity, and students from diverse regions may have marked differences in physical exercise conditions, mobile phone usage habits, and other aspects. Hence, the sample’s geographical distribution covers universities in different regions such as the eastern, central, and western parts, ensuring that the sample has a wide representativeness in terms of geography. This design helps to reveal the potential impact of various regional cultures and economic levels on physical exercise and MPA behavior among college students.
4. School type: to augment the diversity of the study sample, this study chooses a spectrum of university types, encompassing institutions specializing in science and engineering, teacher training, and comprehensive universities. Students from all kinds of universities have various daily course schedules, physical exercise resources, and other aspects. Thus, by selecting multiple types of universities, it is possible to more comprehensively reflect the physical activities and MPA status of students from different backgrounds.

#### 3.2.2 Data collection

Based on the survey design requirements and anticipated statistical analysis methods, this study plans to collect no fewer than 500 valid questionnaires as the analytical sample. Considering potential issues such as incomplete responses or logical inconsistencies, the actual number of distributed questionnaires significantly exceeds the target sample size to ensure final data validity. The 500-sample size guarantees statistical validity and meets the fundamental requirements of various analytical methods including correlation analysis and regression analysis, ensuring good representativeness and analytical stability. The data primarily originates from undergraduate students across multiple Chinese universities, encompassing comprehensive universities, normal universities, and polytechnic institutions from eastern, central, and western regions. This sampling strategy ensures diversity in geographical distribution, academic backgrounds, and educational levels. While specific institution names are withheld due to some universities’ non-disclosure policies, all data collection strictly adheres to academic ethics through voluntary and anonymous participation. To enhance data collection efficiency and accuracy, this study employs a hybrid online-offline questionnaire distribution approach. The online component utilizes survey platforms and student community networks to maximize coverage. In contrast, the offline component involves researchers distributing paper questionnaires during class breaks, in dormitory areas, and at sports facilities, with on-site guidance to ensure response completeness and authenticity. This dual-channel methodology effectively improves sampling efficiency while minimizing potential data errors from survey blind spots or collection biases, thereby providing reliable data support for subsequent analysis.

The online survey used platforms such as Sojump to publish questionnaires. This approach’s advantage lies in that it can collect data quickly and on a large scale, especially in the post-pandemic era, where the convenience and security of online questionnaires have become vital survey methods. The Sojump platform supports access to multiple terminal devices, allowing participants to fill out questionnaires anytime and anywhere through their mobile phones or computers, greatly increasing participation rates. In addition, the questionnaire platform can automatically collect and analyze data, which is helpful for later data management and analysis. During the questionnaire distribution process, links to the questionnaire are posted through internal social media platforms, student clubs, class groups, and other channels, inviting college students to participate in the survey. At the same time, to ensure the participants’ enthusiasm, this study set up a lottery incentive mechanism at the end of the questionnaire to encourage more students to participate in questionnaire filling and ensure the quality of their answers. In terms of offline data collection, the research team has set up dedicated questionnaire collection points on campus and invited students to fill out questionnaires on-site. Offline questionnaire surveys are mainly conducted during breaks or after class, with research team members explaining the research objectives in detail to students to guarantee that they can accurately understand each question when filling out the questionnaire. The collection of offline questionnaires helps to control the survey process, ensure the authenticity and effectiveness of answers, and effectively cover some students who do not often use online tools, supplementing the possible sample bias of online questionnaires. The offline survey is primarily aimed at students who participate in physical education courses or extracurricular physical activities. The research team distributes and collects on-site questionnaires at school playgrounds, gyms, and other locations. This not only ensures the completion rate of the questionnaire but also enables more accurate acquisition of student data on frequent physical exercise.

#### 3.2.3 Data cleaning and processing

After data collection is completed, strict data cleaning and processing are carried out to ensure the questionnaire data’s integrity and accuracy. The concrete steps are as follows:

1. Delete incomplete questionnaires: the submitted questionnaires are screened to remove those that are not filled out, especially those with unanswered key variables, to avoid affecting the accuracy of data analysis (Feng and Sun, 2023).
2. Screen for invalid questionnaires: some participants may answer or resubmit the same questionnaire randomly during the filling process. The research team utilizes consistency checks and time monitoring to eliminate invalid questionnaires that respond too quickly or have contradictory content.
3. Data encoding and input: for completed questionnaires, data encoding is performed and input into statistical software to ensure the operability of subsequent analysis. All questionnaire questions are processed numerically, with variables such as gender and grade encoded accordingly for regression analysis and other statistical processing.

Through the rigorous sample selection and data collection strategies mentioned above, this study ensures the diversity and representativeness of the data. Across the country, factors such as gender ratio, grade distribution, and regional differences have been fully considered (Jiang et al., 2022). The combination of online and offline multi-channel questionnaire distribution methods in the data collection process ensures the efficiency of data collection and increases the sample coverage’s breadth and accuracy. The final collected data undergoes strict cleaning and processing to provide reliable data support for subsequent empirical analysis, ensuring the scientific and accurate nature of the research results (Al-Mamun et al., 2023).

### 3.3 Variable definition and measurement tools

#### 3.3.1 PARS-3

Physical activity rating denotes the comprehensive performance of an individual’s intensity, duration, and frequency of participation in these activities, reflecting their physical exercise levels. To measure college students’ physical activity levels, this study adopts the PARS-3 developed by Japanese scholar Hashimoto (1990) and later revised by Chinese scholar Liang and Liu (1994). This scale has been extensively applied in sports research in multiple countries and regions, with high reliability and validity (Can and Tuna, 2022). PARS-3 evaluates an individual’s physical activity from three dimensions. The first is exercise intensity, which measures the intensity of physical exercise performed by college students. It provides five options, ranging from light exercise (such as walking, doing radio exercises, etc.) to high-intensity endurance exercise (such as running, Pamela high-intensity jumping exercises, etc.). According to the participant’s choice, the exercise intensity score ranges from 1 to 5 points, with higher exercise intensity resulting in higher scores. The second is exercise duration, which evaluates the duration of each workout. The options are classified into 10 min or less, 11–20 min, 21–30 min, 31–59 min, and 60 min or more, with scores of 1, 2, 3, 4, and 5, respectively. The longer the exercise duration, the higher the score. The last is exercise frequency, which measures the number of exercise durations of college students in a certain period. The options are divided into once a month or less (1 point), 2–3 times a month (2 points), 1–2 times a week (3 points), 3–5 times a week (4 points), and almost daily exercise (5 points). The higher the frequency, the higher the score (Hasan et al., 2023).

The scores of these three dimensions are calculated using Equation 1 to determine an individual’s physical activity rating:


(1) $$PA=I\times \left(T-1\right)\times F$$

PA stands for physical activity; I, T, and F refer to exercise intensity, duration, and frequency; higher scores correlate with increased levels of physical activity. The multi-dimensional scale design is instrumental in providing a comprehensive assessment of the physical exercise habits among college students, thereby furnishing foundational data for subsequent analysis.

#### 3.3.2 MPATS

MPAT represents an individual’s excessive dependence on smartphones, including frequent use of mobile phones, psychological dependence on mobile phones, anxiety, and other symptoms without mobile phones (Pearson et al., 2023). To measure college students’ MPAT, this study employs the MPATS compiled by Jie Xiong, Zongkui Zhou, and others. This scale has been extensively applied in the study of college students in China, and has high reliability and validity and structural validity (Abdoli et al., 2023). MPATS consists of 16 questions, classified into four dimensions: withdrawal symptoms, salience behavior, social comfort, and mood changes. Each question adopts the Likert 5-point rating system, with 1 and 5 indicating “strongly disagree” and “strongly agree,” with higher scores demonstrating more severe MPAT. The specific measurement dimensions are as follows:

1. Withdrawal symptom: it measures whether individuals experience discomfort or anxiety during extended periods of mobile phone non-use, as exemplified by the question ‘If I have not used my phone for a long time, I feel uncomfortable’. This dimension reflects individuals’ psychological dependence on mobile phones.
2. Salience behavior: it measures whether an individual frequently uses their mobile phone in daily life, interfering with normal learning or life, such as the question “In class, I may not be able to concentrate because of QQ/WeChat or other social media.” This dimension aims to evaluate the interference of mobile phone usage.
3. Social comfort: it assesses the social comfort derived from mobile phone interactions, exemplified by the query, “I feel more confident when communicating with others on my phone.” This dimension explores the alternative role of mobile phones in social activities.
4. Mood change: the scale appraises the influence of mobile phone usage on emotional states, with a question such as, “When my phone frequently fails to connect to the internet or receive signals, I become anxious and irritable.” This dimension reflects the relationship between mobile phone usage and emotional fluctuations.

The scale is capped at a total score of 80, with higher scores indicative of a more pronounced MPAT. Through the measurement of this scale, the specific performance and severity of MPA in college students can be deeply analyzed, and data support can be provided for follow-up research (Karaoglan Yilmaz et al., 2024).

#### 3.3.3 SAQ

Self-acceptance represents the degree to which an individual recognizes their value, ability, and image, and is a core component of MH (Zwilling, 2022). This study uses the SAQ compiled by Zhong Cong and Wenfeng Gao et al., to measure college students’ self-acceptance levels. The scale has good reliability and validity and is widely adopted to assess an individual’s self-perception and MH status. SAQ involves 16 questions, categorized into self-acceptance and self-evaluation dimensions. The two dimensions include questions with reverse and positive scoring. Each question adopts a 4-point rating system, with 1 and 4 indicating “completely inconsistent” and “completely consistent.” The details of the two dimensions are:

1. Self-acceptance: this dimension encompasses questions such as “I always hesitate to do things because I am afraid of not doing them well” and “I always anticipate failure before doing anything,” mainly reflecting individuals’ negative views on their abilities and personality. The question adopts reverse scoring, that is, a lower score illustrates a higher level of self-acceptance.
2. Self-evaluation: this dimension includes questions such as “I am satisfied with my body and appearance” and “I can do everything well for myself,” used to measure an individual’s affirmation and confidence in their strengths. The scoring for this question is positively oriented, with higher scores reflecting greater self-evaluation levels.

An elevated total score on this scale denotes a higher degree of self-acceptance. By assessing college students’ self-acceptance, the mediating role of their self-perception between physical exercise and MPAT can be elucidated.

#### 3.3.4 CPSS

Perceived stress refers to the level of stress an individual feels in their daily life, typically manifested as emotional tension, a sense of loss of control, and a decreased ability to cope with stress (Yang and Huang, 2003). This study employs the CPSS developed by Tingzhong Yang, Hanteng Huang, and others to measure college students’ stress levels. This scale contains 14 questions, divided into two dimensions: loss of control and tension. Each question adopts a 5-point rating system, where 1 and 5 represent “never” and “almost every day.” An increased score corresponds to a higher level of perceived stress for the individual. The specific dimensions are as follows:

1. Loss of control: this dimension evaluates an individual’s sense of helplessness and powerlessness when dealing with events in life, such as the question “Feeling unable to control important things in their life.” The higher the score, the stronger the individual’s sense of loss of control over important events that occur in life.
2. Tension: this dimension assesses an individual’s emotional response when handling uncertain events, such as the question “Feeling annoyed due to unexpected things.” A higher score indicates a higher individual’s level of tension when facing uncertain events.

The total score of CPSS represents an individual’s overall perceived stress level, with higher scores reflecting increased stress perception. The outcomes derived from this scale offer vital data for examining the correlation between perceived stress and MPA.

## 4 Data analysis and results

### 4.1 Descriptive statistics of the sample

**Figure 1:**
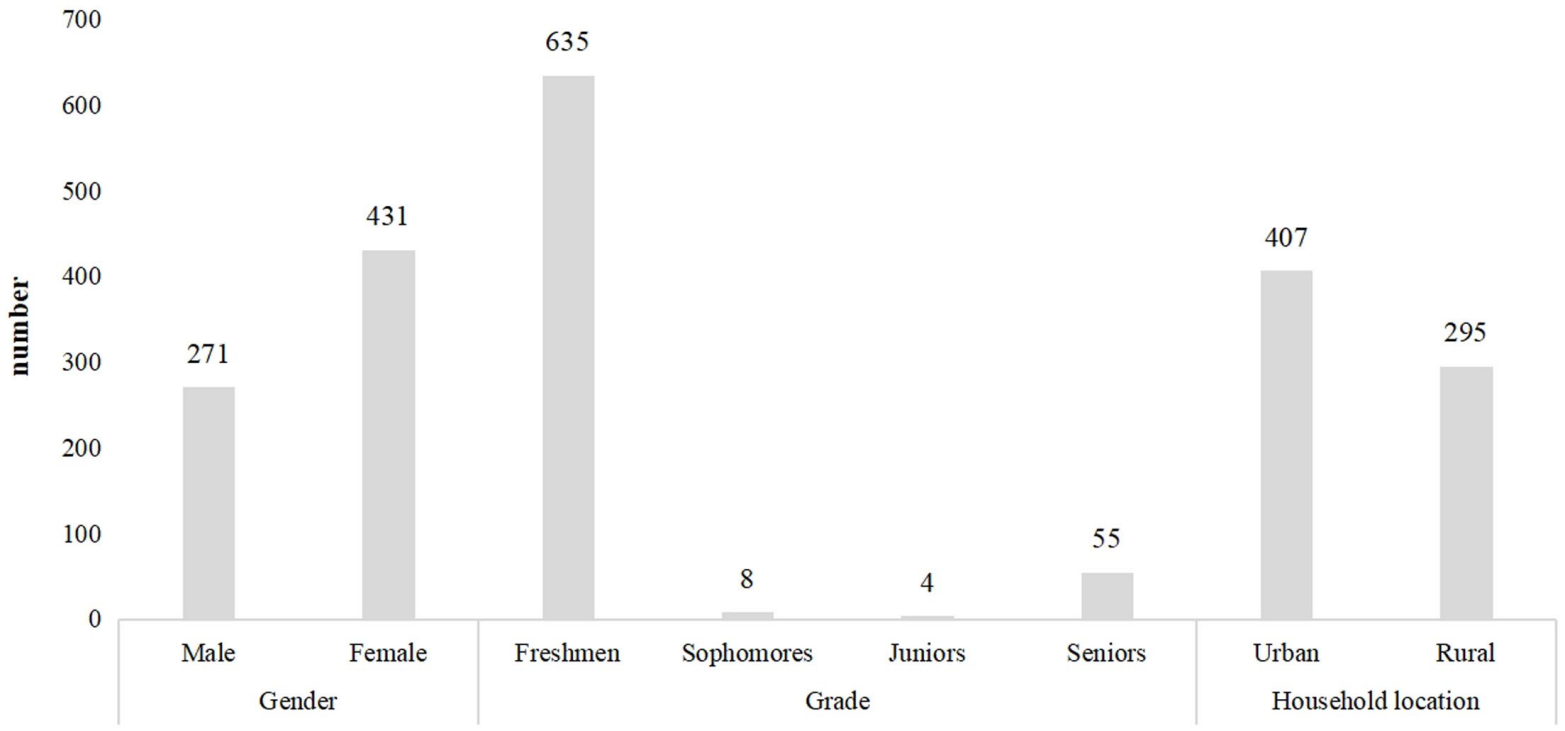
Basic information of participants.

A total of 750 questionnaires were distributed, 723 were recovered, 21 invalid questionnaires were excluded, and 702 valid questionnaires were collected, with an effective recovery rate of 93.6%. The statistical results collected in the first part of the questionnaire are in Figure 1.

Figure 1 shows three basic information of the participants:

1. Gender: male: 271; female: 431.
2. Grade: there are 635 freshmen, 8 sophomores, 4 juniors, and 55 seniors.
3. Household location: the sample comprises 407 and 295 participants from urban and rural regions, respectively.

**Figure 2:**
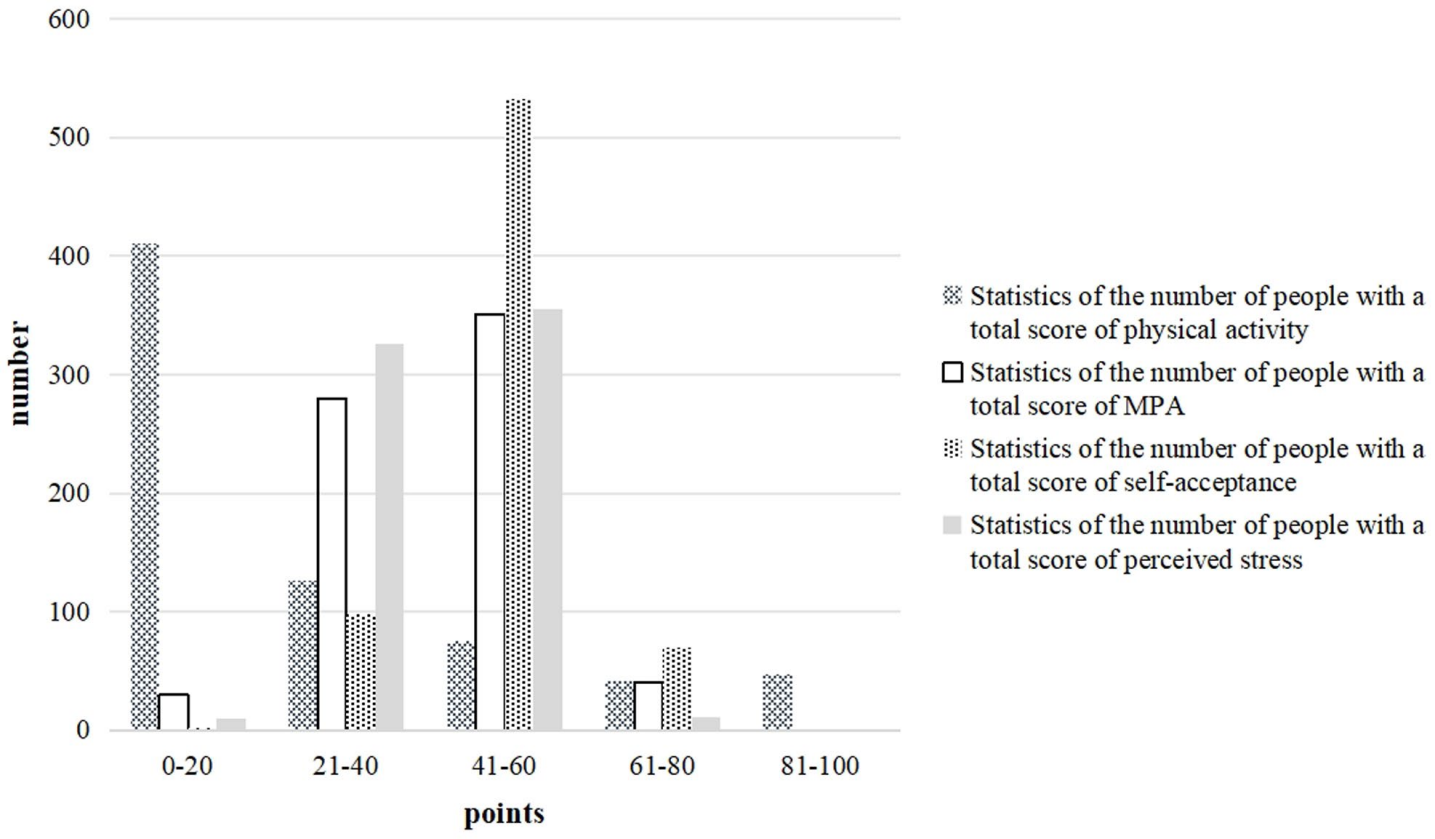
Questionnaire result statistics.

The data from other parts of the questionnaire are sorted through the total score, as exhibited in Figure 2.

Through the organized data, it can be observed that there are 271 male and 431 female students, accounting for 38.6 and 61.4% of the total sample, respectively. This gender ratio shows that female participants account for a larger proportion of the sample, reflecting the bias of the actual survey or the high proportion of females in certain universities. The grade distribution reveals that the majority of participants are freshmen, indicating a high coverage rate of the survey among freshmen. The participation rate of sophomore and junior students is extremely low, which may be due to their academic arrangements, lifestyle habits, and other reasons. The data on the distribution of family locations indicates that urban students account for the majority of the sample. However, the proportion of rural students is relatively large, which can better reflect the differences in physical activities and mobile phone usage habits among college students from different regional backgrounds.

Based on the data in the second part, it can be found that most college students have a low level of physical activity, with over half of them having a physical activity level in the lowest range of “0–20 points.” This illustrates that the majority of participants have a low frequency or intensity of physical exercise, demonstrating that their exercise habits are not strong, and only a small percentage of students (6.7%) have a high level of physical activity (81–100 points). MPAT is mainly concentrated in the range of 21–60 points, with the highest number of participants scoring 41–60 points, accounting for 50%, indicating that half of the students have a moderate level of mobile phone dependence. A very small number of students (5.8%) have a high tendency toward addiction (61–80 points), and only 4.3% of students have a low dependence on mobile phones, in the range of 0–20 points. The self-acceptance level of most students is between 41 and 60 points, reaching 76%, illustrating that the majority of students have a good level of self-acceptance. A very small minority of students (only 0.1%) have a very low level of self-acceptance, while a few students (10%) have a high level of self-acceptance, exceeding 60 points. The perceived stress is mainly concentrated in the range of 21–60 points, especially the 41–60 accounts for about 50.6%, indicating that most students feel moderate stress. A smaller number of students feel lower or higher stress, accounting for 1.4 and 1.6%, respectively.

### 4.2 Correlation analysis between physical exercise and MPA

The experiment conducts a correlation analysis between physical exercise and MPA. By determining the correlation coefficient between the levels of physical activity and the MPA total score, the study can preliminarily assess the impact of physical exercise on mobile phone dependence. The correlation results are outlined in Table 1.

**Table 1:** Correlation analysis.

Dimension	Withdrawal symptom	Salience behavior	social comfort	Mood change
Exercise intensity	−0.32	−0.28	−0.20	−0.30
Exercise duration	−0.25	−0.22	−0.18	−0.27
Exercise frequency	−0.35	−0.30	−0.24	−0.32

In Table 1, exercise intensity presents a moderate negative correlation with all dimensions, especially in withdrawal symptoms (r = −0.32) and mood changes (r = −0.30). It indicates that the higher the intensity of exercise, the less pain and emotional fluctuations students experience when quitting mobile phone use. The correlation between exercise duration and various dimensions is relatively mild, with the highest correlation occurring in mood changes (r = −0.27), indicating that students who exercise for a longer period have relatively stable emotions and fewer negative emotions in MPA. The exercise frequency is the most significant negative correlation factor, with a high correlation with withdrawal symptoms (r = −0.35) and salience behavior (r = −0.30). It illustrates that students who engage in frequent physical exercise exhibit more composure and less impulsive behavior when facing mobile phone withdrawal.

### 4.3 Analysis of the influence of physical exercise on MPA

**Figure 3:**
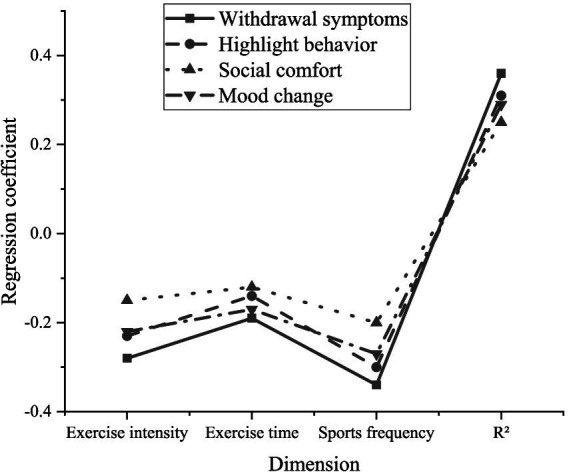
Regression analysis.

To further explore the influence of physical exercise on the MPA behavior of college students, this study analyzes how intensity, duration, and frequency of physical activity affect the four core dimensions of MPA, respectively, through multi-dimensional analysis: salience behavior, withdrawal symptoms, mood change, and social comfort. To this end, the study constructs a multiple regression model to quantify the impact of various dimensions of physical exercise on MPA, generating data to support the analysis. The experimental results are exhibited in Figure 3.

In Figure 3, exercise intensity negatively impacts all dimensions of MPA, especially withdrawal symptoms (*β* = −0.28) and mood changes (*β* = −0.22). This indicates that as the intensity of physical exercise increases, the negative emotional reactions (such as anxiety and tension) of college students facing mobile phone withdrawal can remarkably decrease, and the overall emotional state tends to stabilize. Although the impact of exercise duration on MPA is relatively weak, it still shows a negative correlation in various dimensions. Especially for mood changes (*β* = −0.17) and withdrawal symptoms (*β* = −0.19), extending exercise duration can alleviate emotional fluctuations and reduce discomfort caused by students being unable to use their phones for a long time. The frequency of physical activity is the most significant factor affecting all dimensions of MPA. Particularly in terms of withdrawal symptoms (*β* = −0.34) and salience behavior (*β* = −0.30), frequent exercise can markedly reduce students’ anxiety and strong dependence on mobile phones when they do not have them. In addition, mood changes (*β* = −0.27) also suggest that high-frequency exercise can help students maintain a better emotional state. From the perspective of the overall regression model, the three dimensions of physical exercise have a certain explanatory power for all four aspects of MPA. The highest explanatory effect of the model is for withdrawal symptoms (R^2^ = 0.36), indicating that physical activity has a strong explanatory effect on withdrawal symptoms. However, the explanatory effect for social comfort is relatively low (R^2^ = 0.25), illustrating that physical activity has a relatively small impact on alleviating students’ need for social comfort through mobile phones.

The data analysis reveals that physical exercise significantly mitigates the MPA behavior among college students, particularly frequent physical activity has the most obvious effect on reducing mobile phone dependence. Specifically, frequent exercise can help mitigate negative emotional responses (such as withdrawal symptoms) due to reduced cell phone use and alleviate an individual’s over-reliance on mobile phone behaviors (such as salience behavior).

### 4.4 The moderating effects of self-acceptance and perceived stress on MPA

A hierarchical regression analysis is made based on the data to explore the moderating effects of self-acceptance and perceived stress on physical exercise and MPAT. The moderating effects of different levels of self-acceptance and perceived stress on the four dimensions of MPA (social comfort, mood change, withdrawal symptoms, and salience behavior) are investigated. The concrete results are illustrated in Table 2.

**Table 2:** The moderating effect of self-acceptance and perceived stress.

Dimension	Withdrawal symptom (*β*)	Salience behavior (*β*)	social comfort (*β*)	Mood change (*β*)	R^2^ (No moderating variable)	R^2^ (Moderating variable)
Self-acceptance	−0.30	−0.25	−0.22	−0.28	0.34	0.43
perceived stress	0.35	0.30	0.28	0.32	0.36	0.45
Self-acceptance × Exercise intensity	−0.12	−0.10	−0.09	−0.11	–	–
Perceived stress × Exercise intensity	0.15	0.12	0.10	0.13	–	–

The data in Table 2 demonstrates that students with a higher level of self-acceptance show a stronger mitigation effect on various dimensions of MPA. Self-acceptance is significantly negatively correlated with the four dimensions of MPA, especially in mood changes (*β* = −0.28) and withdrawal symptoms (*β* = −0.30). This illustrates that the higher the level of self-acceptance, the lower the dependence of college students on mobile phones, especially when their phones are not available, showing less emotional fluctuations. In addition, after adding self-acceptance as a moderating variable, the explanatory effect of the model increased from 0.34 to 0.43. It indicates that self-acceptance effectively modulates the relationship between physical activity and MPA, assisting students in further diminishing mobile phone reliance during high-intensity physical activity. Compared with self-acceptance, perceived stress exhibits a significant positive correlation with each dimension of MPA. The higher the perceived stress, the stronger the dependence of students on mobile phones, especially in the dimensions of withdrawal symptoms (*β* = 0.35) and mood changes (*β* = 0.32), where the stress effect is more pronounced. This means that students who perceive higher stress are more likely to show bad emotions such as anxiety and irritability when they are unable to use mobile phones, and MPA behavior is more significant. After adding perceived stress as a moderating variable, the model’s explanatory effect increases from 0.36 to 0.45, indicating that perceived stress exerts a potent moderating influence on the association between physical exercise and MPA. High-level physical exercise can partially alleviate high-stress students’ dependence on mobile phones, but compared to the moderating effect of self-acceptance, the effect is slightly inferior. When the interaction effects of self-acceptance and perceived stress with physical activity are introduced into the model, the data showed that the interaction term of self-acceptance × exercise intensity is negative (*β* = −0.12). It indicates that high-intensity physical activity can further alleviate MPA behavior in students with high self-acceptance, especially in terms of withdrawal symptoms and mood changes. The interaction term of perceived stress × exercise intensity is positive (*β* = 0.15), demonstrating that high perceived stress weakens the alleviating effect of physical exercise on MPA. Even if students participate in high-intensity physical exercise, they may still maintain strong phone dependence due to high pressure.

## 5 Discussion

Descriptive analysis indicates a notably higher proportion of females compared to males. In grade distribution, freshmen dominate the class, while participants from other grades are less. In terms of family location, the proportion of students in urban–rural is relatively balanced. The above distribution characteristics lay a foundation for the subsequent analysis, especially when analyzing the differences between diverse groups, it is necessary to consider the influence of sample distribution. Meanwhile, most college students have a low amount of physical activity, and physical exercise habits are not strong. Among college students, moderate dependence on MPA accounts for the largest proportion, indicating that half of the students have a certain degree of dependence on mobile phones, but the situation of excessive addiction is less. The overall level of self-acceptance is good, and students generally have a high degree of self-recognition and acceptance. The perceived stress of most students is at the medium level, but a few students feel greater stress. These data provided a basis for subsequent analysis of the relationship between physical activity, MPA, self-acceptance, and perceived stress. The results of correlation analysis reveal that the intensity, duration, and frequency of physical activity are negatively correlated with the four dimensions of MPA, especially the exercise frequency has the most significant effect on reducing mobile phone dependence. The higher the exercise intensity, duration, and frequency, the more stable the performance of college students in four aspects of mobile phone withdrawal symptoms, salience behavior, social comfort, and mood change, and the lower the dependence on mobile phones.

Regression analysis shows that the frequency, intensity, and duration of physical activities have varying degrees of negative effects on MPA among college students, with an increase in frequency having the most significant impact on withdrawal symptoms and salience behavior. These findings provide empirical support for promoting physical exercise as an effective strategy for intervening in MPA in universities in the future. Regular physical exercise not only enhances physical fitness but also aids college students in better managing their mobile phone usage, mitigating the negative emotions and social issues stemming from mobile phone dependence. In multi-layer regression analysis, the higher the level of self-acceptance, the better students can resist the negative emotions brought by MPA, especially in high-intensity physical activities. Physical exercise can help students with higher levels of self-acceptance further reduce their dependence on mobile phones. Moreover, perceived stress is a vital risk factor, with students with higher levels of perceived stress exhibiting more severe MPA behavior. Even high-intensity physical exercise cannot completely offset this risk, especially when faced with withdrawal symptoms from mobile phones, high-stress students still exhibit significant emotional fluctuations and dependency behaviors.

## 6 Conclusion

A series of valuable conclusions are obtained through the in-depth study of the relationship between college students’ physical exercise and MPA. These conclusions not only reveal the potential role of physical activities in improving MPA behavior but also provide feasible intervention measures for health education in universities. Combining the sample data analysis and theoretical model, the following conclusions can be drawn:

Physical exercise has a significant effect on reducing MPA among college students. Exercise intensity, duration, and frequency show significant negative correlations with various dimensions of MPA (withdrawal symptoms, salience behavior, social comfort, and mood change). Especially, exercise frequency has a distinct effect on reducing withdrawal symptoms and highlighting behaviors. Frequent physical exercise can effectively alleviate students’ anxiety and irritability when they cannot use their phones, and reduce their phone dependence behavior. Self-acceptance, as an important psychological factor, plays a remarkable moderating effect between physical exercise and MPA. The study depicts that students with higher levels of self-acceptance have markedly reduced MPAT, especially in the face of mobile phone withdrawal symptoms and mood changes. Self-acceptance not only directly reduces the dependence on mobile phones but also further plays a role in alleviating MPA in high-intensity physical exercise. Compared with self-acceptance, perceived stress, as a critical risk factor, is significantly positively correlated with MPA. Students with higher perceived stress had more prominent negative performance in withdrawal symptoms and mood changes. Although physical exercise can alleviate such negative effects to a certain extent, the positive effect of physical activity is weakened under high-stress conditions. Therefore, stress management is essential to reduce MPA. The study further elucidates that self-acceptance and perceived stress exert a significant interactive effect on the nexus between physical exercise and MPA. Students possessing elevated levels of self-acceptance derive greater advantages from high-intensity physical exercise, resulting in a pronounced reduction in MPAT. Students with higher perceived stress are likely to maintain a strong mobile phone dependence, even if they participate in high-frequency, high-intensity physical activities. These results suggest that psychological factors play a vital moderating role in the influence of physical exercise on MPA.

This study empirically examines the impact of physical exercise on college students’ MPA and incorporates psychological variables such as self-acceptance and perceived stress for mediating mechanism analysis. However, certain limitations exist in research design and data interpretation. First, the sample structure exhibits bias. Although the methodology section describes quality control measures, the collected questionnaires show significant imbalances in gender and grade distribution. Female participants (*n* = 431) substantially outnumber males (*n* = 271), while freshmen constitute an absolute majority. The research design implements quality controls through hybrid online-offline data collection across multiple universities with multi-grade participation incentives. However, operational realities reveal higher response rates among freshmen, compounded by onsite surveys primarily conducted in physical education classes and extracurricular venues, resulting in observable gender and grade disparities. While these biases partially reflect actual participation patterns, it is acknowledged that unbalanced sample composition may affect the generalizability and explanatory power of research results. Especially for senior students or groups with different professional backgrounds, there may be differences in exercise participation, psychological stress, and mobile phone use behaviors. Future studies could refine sampling strategies to achieve better balance across gender, grade, and academic disciplines while strengthening monitoring of external variables (e.g., major events) to enhance result robustness. Additionally, detailed disciplinary background analysis is lacking, though significant differences in psychological states, exercise participation, and mobile phone behaviors across majors warrant further investigation. Second, insufficient control exists for external variables and major events. The study does not account for potential confounding effects of significant external events (e.g., pandemics, exam periods) that might influence MH or mobile phone usage behaviors. These events possibly introduce interference in variable relationships and compromise conclusion accuracy and stability. Third, regarding the verification of causal relationships, this study employs a cross-sectional survey design during the research phase, primarily considering its advantages in broad sample coverage and high data collection efficiency. This approach facilitates the rapid acquisition of large-scale data across multiple universities, enabling a comprehensive examination of the relationships between physical exercise, self-acceptance, perceived stress, and MPA. In data analysis, multiple regression, interaction term modeling, and others are utilized to preliminarily explore potential pathways among variables and establish a theoretical framework for causal relationships. However, it demonstrates the inherent limitations of cross-sectional studies in causal inference. Without longitudinal tracking or randomized interventions, the current design cannot provide conclusive causal evidence. The present findings primarily represent “associational discoveries.” Future research could adopt more rigorous experimental designs or longitudinal approaches to more scientifically validate the causal effects of physical exercise on smartphone addiction behaviors. Potential methodological improvements include implementing exercise interventions, establishing control and experimental groups, and conducting time-series analyses for enhanced causal inference. Moreover, the study incorporates mechanisms for controlling external variables (e.g., examination periods, major social events). Also, it expands the diversity of cultural backgrounds and individual characteristics within samples to enhance the explanatory power and generalizability of findings. The samples in this study are mainly from some universities in China. The potential influence of different cultural contexts, educational environments, or developmental experiences on students’ physical exercise behavior and mobile phone dependence levels has not been thoroughly considered. The external applicability and generalization of the research results are still limited.

Through the above optimization path, future research could be more helpful to clarify the deep relationship between physical exercise and MPA, thus enriching the theory and practice system of behavior intervention and psychological adjustment.

## Data Availability

The original contributions presented in the study are included in the article/Supplementary material, further inquiries can be directed to the corresponding author.
