# Supplementary material for: The impact of physical exercise on mobile phone addiction among college students: a study based on Chinese universities
Source: Front Psychol. 2025 Apr 14;16:1524520. doi: 10.3389/fpsyg.2025.1524520 (PMC12034732; doi:10.3389/fpsyg.2025.1524520)
Supplement: Supplementary file 1 [file Supplementary_file_1.docx]

**Questionnaires of the Investigation of College Students' Physical Exercise and Mobile Phone Dependence**

**Part 1: Basic information**

1. Gender: Male 1; Female 2

2. Grade: Freshman 1; Sophomore 2; Junior 3; Senior 4

3. Household location: Urban Region 1; Rural Region 2

**Part 2: The Physical Activity Rating Scale (PARS-3)**

The study employs the Physical Activity Rating Scale (PARS-3) originally developed by Japanese scholar Kimio Hashimoto (1990) and later revised by Chinese scholar Deqing Liang (1994). This scale evaluates individuals' physical activity levels across three dimensions: exercise intensity, duration, and frequency, each scored on a 1-5 scale. The final physical activity score is calculated using the equation: Physical Activity Level = Intensity × (Duration - 1) × Frequency, with higher scores indicating greater physical activity engagement. [Reference: Deqing Liang. Stress levels among college students and their relationship with physical exercise. Chinese Mental Health Journal, 1994(01):5-6.]

**Specific questions of PARS-3:**

1. What is the intensity of your physical exercise? (Single choice, scored 1-5: A=1, E=5)

A. Very light (e.g., walking, radio calisthenics)

B. Light but not intense (e.g., jogging, tai chi)

C. Moderately intense and sustained (e.g., running, table tennis)

D. High intensity with heavy breathing/sweating but not sustained (e.g., badminton, basketball)

E. High intensity with heavy breathing/sweating and sustained (e.g., racing, Pamela Reif high-intensity interval training)

2. How long does one session of your physical activity typically last (including physical education classes)? (Single choice, scored 1-5: A=1, E=5)

A. <10 minutes

B. 11-20 minutes

C. 21-30 minutes

D. 31-59 minutes

E. ≥60 minutes

3. How frequently do you engage in physical activity? (Single choice, scored 1-5: A=1, E=5)

A. ≤1 time/month

B. 2-3 times/month

C. 1-2 times/month

D. 3-5 times/month

E.  Nearly daily

4. Which exercise type do you prefer? (Single choice, non-scored categorical variable)

A. Aerobic

B. Anaerobic

C. Both equally

5. What specific sports do you enjoy? (Multiple choice, non-scored categorical variables)

A. Track & field (sprinting, jogging, marathon, long jump, high jump)

B. Team ball sports (football, basketball, volleyball)

C. Racket sports (badminton, table tennis, tennis, baseball)

D. Leisure activities (walking, yoga, dance sports)

E. Outdoor (hiking, mountaineering, cycling)

F. Indoor (machine training, strength training)

G. Other _______________

**Part 3: The Mobile Phone Addiction Tendency Scale (MPATS) (5-point scale: 1 = Strongly disagree, 5 = Strongly agree)**

The Mobile Phone Addiction Tendency Scale (MPATS), developed by Jie Xiong, Zongkui Zhou et al., consists of 16 items using a 5-point Likert scale ranging from "strongly disagree" to "strongly agree." It comprises four dimensions: withdrawal symptoms (items 1,4,6,8,10,12), salience behavior (items 5,9,13,15), social comfort (items 2,7,16), and mood change (items 3,11,14). Scores range from 16 to 80, with higher scores indicating greater mobile phone dependency. Scores below 47 represent normal usage, while 48 and above suggest dependency. Exploratory and confirmatory factor analyses demonstrate good reliability and validity among Chinese college students, with an internal consistency coefficient of 0.83 and test-retest reliability of 0.91. [Reference: Jie Xiong, Zongkui Zhou, et al. Development of the Mobile Phone Addiction Tendency Scale for College Students. Chinese Mental Health Journal, 2012,26(03):222-225.]

**Specific questions of MPATS:**

1. If I don't have my phone with me for a while, I immediately check for new messages or missed calls.

2. I prefer chatting on my phone over face-to-face communication.

3. While waiting for someone, I frequently send messages or call to ask where they are—without doing so, I feel restless.

4. I feel uncomfortable if I haven't used my phone for a long time.

5. In class, I struggle to focus on lectures because of QQ/WeChat or other social apps.

6. I feel lonely without my phone.

7. I feel more confident when communicating with others via my phone.

8. If my phone doesn't ring for a while, I feel uneasy and instinctively check for missed calls or new messages.

9. I often experience the illusion that "my phone is ringing or vibrating."

10. Having many calls or messages makes me feel more fulfilled.

11. I often worry about my phone shutting down automatically.

12. My phone feels like a part of me; reducing usage makes me feel like I've lost something.

13. My classmates/friends/family often say I rely too much on my phone.

14. Poor internet connection or weak signal makes me anxious and irritable.

15. In class, I frequently focus on my phone instead of listening to the lecture.

16. I find it more comfortable to interact with others through my phone.

**Part 4: Self-Acceptance Questionnaire (SAQ)**

The Self-Acceptance Questionnaire (SAQ), developed by Zhong Cong and Wenfeng Gao, contains 16 items divided into two 8-item dimensions: self-acceptance and self-evaluation, using a 4-point scale. Higher total scores indicate greater self-acceptance. The self-acceptance dimension (items 1,4,7,8,11,13,14,16) uses reverse scoring, while the self-evaluation dimension (items 2,3,5,6,9,10,12,15) employs positive scoring. The questionnaire demonstrates good reliability with a total α coefficient of 0.76 and subscale coefficients of 0.91 and 0.90. [Reference: Zhong Cong and Wenfeng Gao. The development of self-acceptance questionnaire and the test of its reliability and validation. Chinese Journal of Behavioral Medicine and Brain Science, 1999(01):20-22.]

**Specific questions of SAQ:**

1. I never dare to voice my inner desires.

2. I possess almost entirely strengths and advantages.

3. I believe people of the opposite sex would definitely like me.

4. I avoid attempting tasks for fear of failure.

5. I am very satisfied with my physique and appearance.

6. Overall, I am quite satisfied with myself.

7. I only feel assured when others approve of my actions.

8. I constantly worry about criticism or blame from others.

9. I learn new things faster than others.

10. I am very satisfied with my eloquence.

11. I always anticipate failure before doing anything.

12. I can handle all my tasks competently.

13. I think nobody likes me.

14. I fear upsetting others.

15. I genuinely like my personality traits.

16. I worry others look down on me.

**Part 5: The Chinese Perceived Stress Scale (CPSS) (5-point scale: 1 = Never, 5 = Almost daily)**

The Chinese Perceived Stress Scale (CPSS), developed by Tingzhong Yang and Hanteng Huang, consists of 14 items across two 7-item dimensions: loss of control (items 4,5,6,7,9,10,13, reverse-scored) and tension (items 1,2,3,8,11,12,14, positively scored). Using a 5-point scale, higher total scores indicate greater perceived stress. [Reference: Tingzhong Yang and Hanteng Huang. An epidemiological study on stress among urban residents in social transition period. Chinese Journal of Epidemiology, 2003(09):11-15.]

**Specific questions of CPSS:**

1. I get upset because of unexpected events.

2. I feel unable to control important things in my life.

3. I feel nervous or stressed.

4. I successfully handle irritating matters in life.

5. I feel capable of managing important life changes effectively.

6. I feel confident about solving my problems.

7. I feel things are going as I expected.

8. I find myself unable to handle all the things I need to do.

9. I have ways to control annoying things in life.

10. I feel in control of my life.

11. I get angry when things happen beyond my control.

12. I often think about things I must accomplish.

13. I can manage my time well.

14. I often feel difficulties pile up beyond my ability to overcome.
